# Identification of cognitive predictors of remission in depression following limited effect of repetitive transcranial magnetic stimulation on hot and cold cognitive systems

**DOI:** 10.3389/fnhum.2025.1696560

**Published:** 2025-12-17

**Authors:** Dorsa Derakhshan, Nir Lipsman, Anthony Feinstein, Anthony Levitt, Moshe Eizenman, Jennifer Rabin, Peter Giacobbe

**Affiliations:** 1 Harquail Centre for Neuromodulation, Sunnybrook Research Institute, Toronto, ON, Canada; 2 Institute of Medical Science, Temerty Faculty of Medicine, University of Toronto, Toronto, ON, Canada; 3 Department of Surgery, Temerty Faculty of Medicine, University of Toronto, Toronto, ON, Canada; 4 Department of Psychiatry, Temerty Faculty of Medicine, University of Toronto, Toronto, ON, Canada; 5 Department of Biomedical Engineering, University of Toronto, Toronto, ON, Canada

**Keywords:** major depressive disorder, rTMS, cognitive dysfunction, DLPFC, attentional bias, eye tracking

## Abstract

**Introduction:**

Cognitive dysfunction is a chronic and debilitating element of major depressive disorder (MDD), which manifests as abnormal processing in hot (emotion-laden) and cold (emotion-independent) cognitive systems. Although the antidepressant properties of repetitive transcranial magnetic stimulation (rTMS) are well-established, its impact on hot and cold cognition requires further elucidation.

**Methods:**

Prospective study of patients with MDD undergoing an acute course of high frequency rTMS to the left dorsolateral prefrontal cortex (L-DLPFC). MDD patients (*N* = 24) received a 4-to-6-week course of rTMS during which they were evaluated for depressive symptoms and completed cognitive assessments. Age-, sex-, and education-matched healthy controls (*N* = 33) also completed the cognitive tasks at the same intervals as the MDD patients.

**Results:**

Sustained antidepressant effect was observed following rTMS in MDD patients. Hot and cold cognition remained unaltered over the course of treatment. A pre-treatment baseline cognitive phenotype of those who achieve remission of their depressive symptoms with rTMS was identified, characterized by greater sustained attention, speed in correct identification of facial expressions, and free recall of words.

**Conclusion:**

Our findings further validate the cognitive safety and clinical efficacy of rTMS as an intervention for MDD. Future research is required to further characterize the utility of pre-rTMS cognitive phenotyping in identified remitters, to aid in patient selection and treatment prognostication.

## 1 Introduction

The World Health Organization has identified depressive disorders, including Major Depressive Disorder (MDD) as one of the major leading causes of disability worldwide, affecting approximately 332 million individuals (World Health Organization, 2025). A variety of evidence-based therapeutic options are currently available to MDD patients, including traditional treatments such as pharmacotherapy and psychotherapy, as well as alternative and novel approaches including neurostimulation interventions (Milev et al., 2016). While pharmacotherapy is considered a first-line treatment, approximately one third of MDD patients suffer from suboptimal response or complete nonresponse to prescribed medications – a condition referred to as treatment-resistant depression (TRD) (Ionescu et al., 2015; Kautzky et al., 2017). TRD is characterized by the failure to achieve clinical response or remission of symptoms following at least 2 trials of antidepressant medication of adequate dosage and duration (Fava, 2003; Ionescu et al., 2015; Rybak et al., 2021; Thase and Rush, 1995). Patients with TRD often report greater illness severity and chronicity as well as poor response to prescribed treatments (Kornstein and Schneider, 2001).

According to the Diagnostic and Statistical Manual of Mental Disorders, Fifth Edition, Text Revision (DSM-5-TR), cognitive dysfunction is considered to be a criterion of MDD (American Psychiatric Association, 2022; Cohen et al., 2012). The cognitive domains most commonly implicated in those presenting with MDD are executive functioning, working and verbal memory, and attention and processing speed (McIntyre et al., 2013; Woo et al., 2016), and these areas remain compromised beyond clinical remission (Ardal and Hammar, 2011; Conradi et al., 2011; Hammar et al., 2010; Hasselbalch et al., 2011; Herrera-Guzmán et al., 2010; Lam et al., 2014; Woo et al., 2016). Various factors have been shown to influence cognitive function in those with MDD, including sex (Wang et al., 2020; Zhao et al., 2021), old age (Gorwood et al., 2008; Thomas et al., 2009), later age of onset of MDD (Bora et al., 2013; Herrmann et al., 2008; Wekking et al., 2012), lower education level (Baune et al., 2009; Gildengers et al., 2012; Gorwood et al., 2008; Lee et al., 2012), and childhood adversity (Lemos-Miller and Kearney, 2006; Riso et al., 2002). Disorder-related characteristics are also associated with greater cognitive dysfunction such as the usage of prescribed medication shown to influence alertness and cognitive flexibility, duration of current depressive episode, and greater illness severity (Schwert et al., 2019). In recent years, cognitive dysfunction has received increasing recognition as a cardinal and persisting component of MDD, with a reported prevalence of 9 in 10 patients during active depressive episode, even persisting in 4 in 10 patients following remission (Conradi et al., 2011; Pu et al., 2018). While the exact prevalence of cognitive dysfunction in depression remains unknown (Clark et al., 2016; Pu et al., 2018), it is a key determinant of functional outcome (Gupta et al., 2013; Mrazek et al., 2014) and imposes persistent debilitating effects on personal and societal levels (McIntyre et al., 2013; Petersen et al., 2004; Roiser and Sahakian, 2013).

Several cognitive models have been proposed to conceptualize cognitive dysfunction in MDD. These include Beck’s cognitive model of depression (Beck, 2008), Harmer’s theory on the neuropsychopharmacological basis of cognition in MDD (Harmer et al., 2009; Harmer and Cowen, 2013), and Rosier and Sahakian’s hot and cold cognitive systems (Roiser and Sahakian, 2013). According to Beck’s cognitive model, biased information processing gives rise to the emergence and maintenance of major depression, perpetuated by stressors, personality traits, early adverse events, and genetic predisposition, and include inhibitory cognitive deficits involving the inability to disengage from negative stimuli, ruminative response style, negatively biased attention, memory and attitudes, and depressive self-referential schemas (Beck, 1967; Beck, 2008; Disner et al., 2011; Gotlib and Joormann, 2010; Joormann and Gotlib, 2006). The association between cognitive dysfunction and abnormal emotional processing has been central to Beck’s cognitive theory of MDD as it regards mood-congruent bias as a prominent feature of depression, affecting various cognitive functions such as attention, memory, and reasoning faculties (Beck et al., 1979). The cognitive neuropsychological theory of antidepressant mechanism of action explains the role of pharmacological and psychological treatment in changing negative cognitive biases in MDD, through bottom-up and top-down effects, respectively (Godlewska and Harmer, 2021; Harmer et al., 2009; Roiser et al., 2012; Roiser and Sahakian, 2013). It proposes that antidepressants influence cognition initially through promoting a gradual attenuation of biased bottom-up processing of affective stimuli, thereby increasing positive emotional processing, with these effects preceding mood improvement (Clark et al., 2009; Godlewska and Harmer, 2021; Harmer et al., 2009; Robinson and Sahakian, 2008). This model provides a plausible explanation for the observed delay in clinical improvement following pharmacotherapy as it includes the additional time required to witness implicit and immediate changes on a neuronal level in conscious emotional processing (Harmer and Cowen, 2013; Roiser et al., 2012). A recent model by Roiser and Sahakian (2013) conceptualizes cognitive dysfunction in MDD as two distinct domains implicated in the pathophysiology of this condition, termed “hot” (emotion-laden or mood-congruent) and “cold” (emotion-independent) cognitive systems (Roiser and Sahakian, 2013). Titled as the cognitive neuropsychological model of depression, it visualizes the dysregulation of hot and cold cognitions through the abnormal activation patterns of their mutually reinforcing networks – specifically the hyperactivation of subcortical structures and the hypoactivation of cortical areas (Hopman et al., 2021; Roiser and Sahakian, 2013). Limbic and subcortical regions’ hyperactivity – including the amygdala, nucleus accumbens, and the subgenual anterior cingulate cortex (sgACC) – and the compromised monoaminergic system which regulates these areas result in aberrant “bottom-up” processing, leading to the genesis of maladaptive negative perceptions in depression, while hypoactivity of cortical regions involved in cold cognition, including the dorsolateral prefrontal cortex (DLPFC), dorsal ACC, and the hippocampus, may cause cold cognitive system deficits, cognitive biases, and negative expectations (Hopman et al., 2021; Roiser and Sahakian, 2013). Given that neurobiological substrates are at the foundation of hot and cold cognition framework, exploring this integrative model is relevant in brain stimulation research in its clinical predictive value and developing precision medicine approaches in MDD (Dam et al., 2021).

Available treatments for cognitive dysfunction in depression include selective serotonin reuptake inhibitor (SSRI) and serotonergic-noradrenergic reuptake inhibitor (SNRI) antidepressants which have shown to improve cognition independently from their positive mood-related impact, with procognitive effects in various domains of executive function, attention, processing and psychomotor speed, memory acquisition, and episodic and working memory (du Jardin et al., 2014; Herrera-Guzmán et al., 2009; McIntyre and Lee, 2016). An example is the antidepressant Vortioxetine which has demonstrated procognitive effects that are direct and independent from its therapeutic response (Bennabi et al., 2019; Mahableshwarkar et al., 2015; McIntyre et al., 2014; McIntyre et al., 2016; Zuckerman et al., 2018). Considering the limitations of pharmacological treatment, particularly the probability of suboptimal or complete non-response in MDD, neurostimulation interventions have provided a novel path to clinical improvement.

Repetitive transcranial magnetic stimulation (rTMS) is a non-invasive, tolerable, and safe alternative treatment for MDD which generates electrical activity within the brain’s neural tissue by utilizing an inductor coil against the scalp as it produces robust and focal magnetic field pulses (Burke et al., 2019; Hallett, 2007; Milev et al., 2016). High frequency (HF) rTMS over the left dorsolateral prefrontal cortex (L-DLPFC) has shown to improve depressive mood symptoms (Pascual-Leone et al., 1996). Various parameters can be adjusted to optimize rTMS for delivering personalized, patient-centred care including stimulation intensity, pattern (e.g., standard rTMS or intermittent theta burst stimulation (iTBS)), frequency (high or low), and target site most commonly being the DLPFC (Burke et al., 2019; Milev et al., 2016). High frequency (HF) rTMS (5-20 Hz) elicits excitatory effects while low frequency (LF) rTMS (1-5 Hz) is inhibitory (Burke et al., 2019; Milev et al., 2016). It has been proposed that the antidepressant efficacy of DLPFC-rTMS occurs along with its direct effects on cognitive processes involved in emotion regulation since it aims at the DLPFC node of the cognitive control network (Lantrip et al., 2017). In addition, studies have identified potential neurophysiological mechanisms of rTMS, highlighting the aberrant electroencephalography (EEG) signaling in MDD patients and their observed modulation following rTMS in resembling patterns similar to healthy individuals (Ebrahimzadeh et al., 2021; Ebrahimzadeh et al., 2023; Ebrahimzadeh et al., 2024; Nikravan and Ebrahimzadeh, 2021). The safety of rTMS has been well-validated by the literature, as the most commonly reported adverse events (AEs) are transient headache or neck pain, while the serious, but notably rare, AE associated with this treatment is the induction of seizure (Burke et al., 2019). Current evidence for the cognitive effects of L-DLPFC rTMS supports the absence of deleterious cognitive side-effects, validating the cognitive safety of this treatment (Blumberger et al., 2018; Hopman et al., 2021; Kaster et al., 2018; Lantrip et al., 2017; Milev et al., 2016; Miron et al., 2019; Schulze et al., 2016; Serafini et al., 2015), however, the effects of rTMS on hot and cold cognition remain to be fully elucidated.

Empirical evidence, including large-scale meta-analyses, report inconclusive findings for the effects of rTMS on hot and cold cognitive systems which may be the result of substantial heterogeneity in research design (Burke et al., 2019; Fu et al., 2025; Guse et al., 2010; Martin et al., 2017; Mutz et al., 2023; Serafini et al., 2015; Toffanin et al., 2022). For instance, no changes in cognitive processes of attention, short-term memory, and executive function were found following unilateral and bilateral rTMS (Blumberger et al., 2012). There have also been contrary results for deep transcranial magnetic stimulation (dTMS) (Harel et al., 2014; Levkovitz et al., 2009). While some studies propose negligible or no influence of rTMS on cold cognition in MDD (Fitzgerald et al., 2006; Fu et al., 2025; Kaster et al., 2018; Loo et al., 2001; Moreines et al., 2011), others have reported improvement in executive function, working, verbal, and visuospatial memory, sustained attention, processing and psychomotor speed, and response inhibition (Asgharian Asl and Vaghef, 2022; Asgharian Asl et al., 2024; Begemann et al., 2020; Göke et al., 2025; Harvey et al., 2015; Kedzior et al., 2012; Lantrip et al., 2017; Levkovitz et al., 2009; Martis et al., 2003; Monira et al., 2020; Rostami et al., 2022; Tong et al., 2021). Recent research led by Hopman et al. (2021) also used the Cambridge Neuropsychological Test Automated Battery (CANTAB) and reported improvement in target detection sensitivity in sustained attention and efficiency of executive functioning (Hopman et al., 2021). The normalization of hot cognition in the domains of attention and processing speed has also been noted (Moreines et al., 2011; Vanderhasselt et al., 2009b). These proposed procognitive effects have been observed following both a single session as well as a complete 30-visit rTMS treatment course (Corlier et al., 2020; Luber and Lisanby, 2014; Vanderhasselt et al., 2007; Vanderhasselt et al., 2009a). While an association between clinical improvement and greater cognitive performance has been found (Corlier et al., 2020), this observation remains unclear as the cognitive enhancements post-rTMS have also been suggested to be independent from improved mood (Martin et al., 2017; Martis et al., 2003). Although the literature presents promising findings on this topic, the connection between cognition and antidepressant outcome of rTMS and the underlying neurological mechanisms have yet to be clarified (Asgarinejad et al., 2024). Given the crucial role of cognition and functional recovery in patients’ quality of life, exploring the impact of rTMS on cognitive dysfunction in MDD is of paramount significance for the wellbeing of both active and remitted MDD patients (Pan et al., 2019; Woo et al., 2016). This study contributes to the emerging literature on rTMS for depression and its impact on cognition.

The primary aim of this study was to investigate whether changes in cognition occur over the course of rTMS and assess the time course of these changes relative to antidepressant effects.

Our primary hypotheses of this study were as follows:

*H1a*: Responders (defined as patients experiencing ≥ 50% reduction in HDRS-17 at end of treatment (i.e., Week 6) relative to their pre-rTMS baseline) will demonstrate significant change in hot cognition in comparison to non-responders, as assessed by a reduction in average fixation time on dysphoric images.

*H1b*: Responders will demonstrate comparable patterns of attentional bias toward emotional laden stimuli as healthy controls.

*H2a*: Responders will exhibit significant improvement in cold cognition in comparison to non-responders, as assessed by executive function.

*H2b*: Responders will exhibit comparable patterns of executive functioning as healthy controls.

*H3*: Any improvements in hot and cold cognition will precede antidepressant effects assessed by HDRS-17 and QIDS-16SR.

Our secondary hypothesis was that the therapeutic antidepressant outcomes of dTMS and iTBS protocols will be comparable, as assessed by HDRS-17 and QIDS-16SR.

## 2 Materials and methods

### 2.1 Study design

This study was a prospective, open-label, single-site project based at the Harquail Centre for Neuromodulation at Sunnybrook Health Sciences Centre, Toronto, Ontario, Canada. Eligible MDD outpatients received one of two Health Canada-approved L-DLPFC HF-rTMS protocols for MDD, dTMS and iTBS, of their choosing. Both protocols were delivered 5 days per week for up to 6 weeks, for a total of 30 treatment sessions. For safety considerations, information on incidents of AEs were collected at each visit following screening visit. Patients completed the 16-item self-rated Quick Inventory of Depressive Symptomatology (QIDS-16SR) for mood self-assessment on a weekly basis (Rush et al., 2003), while the physician-rated 17-item Hamilton Depression Rating Scale (HDRS-17) (Hamilton, 1960) for clinical assessment, CANTAB cognitive battery, and the eye tracking task were administered biweekly at five timepoints of Week 0 (baseline), Week 2, Week 4, Week 6 (final visit), and Week 10 (follow-up). For normative comparison of clinical symptomatology and cognitive performance, healthy controls also completed the QIDS-16SR, CANTAB cognitive battery, and the eye tracking task at three time points of Week 0, Week 2, and Week 6 (refer to Supplementary Figure 1. Study Timeline Overview for an overview of study measures across time and Supplementary Figure 2. CONSORT Flow Diagram for a visual representation of participant involvement from enrolment to final analysis).

### 2.2 Participant selection

Inclusion criteria were: adults between ages of 18 and 65, met DSM-5 criteria for MDD diagnosis determined by the Mini-International Neuropsychiatric Interview (MINI), baseline HDRS-17 score of greater than or equal to 16, capable of providing written informed consent, and stable dosage of antidepressants for a minimum of 4 weeks prior to rTMS treatment initiation for patients who were using psychiatric medication. Exclusion criteria were: unstable medical conditions, current or past history of epilepsy, current or past diagnosis of an anxiety disorder, bipolar disorder, schizophrenia, and psychotic disorders (such as substance-induced psychosis, psychotic disorder not otherwise specified, and psychotic disorder due to general medical condition) as confirmed by the MINI, recent history of substance dependence and/or abuse within the past 6 months as outlined by DSM-5 (excluding nicotine and caffeine), history of suicide attempt within the past 12 months, past non-response to rTMS, ECT completion within the past 3 months, and limited English language proficiency.

For the healthy control group, eligible participants were to be devoid of current or past history of neuropsychiatric illness as determined by a negative screen for past, current, or recurrent depression, suicidality, generalized anxiety, phobias, mania, and post-traumatic stress disorder on the MINI. Healthy controls were age-, sex-, and education-matched to the MDD group. *A priori* analysis for sample size considerations with StudySize 2.0 program used a repeated measures design with two groups and six repeated measures assuming two-sided alpha 0.05 and a statistical power level of 0.8 On the basis of detecting a 4-s reduction in average fixation time between responders and non-responders (Eizenman et al., 2003), we aimed to enroll 70 participants and allow for an estimated 20% drop out rate in order to reach 56 participants as final sample size.

### 2.3 rTMS treatment

#### 2.3.1 Motor threshold (MT) evaluation and coil placement

Resting motor threshold (RMT) is defined as the minimum dosage of magnetic energy delivered in a single pulse to the motor cortex capable of stimulating motor neuron activity and maximal contralateral thumb muscle (pollicis brevis) contraction during at least 50% of stimulations (Westin et al., 2014). RMT was determined through visual inspection following the positioning of the coil at 45 degrees over the target site (Burke et al., 2019; Harel et al., 2011; Milev et al., 2016). Subsequently, the coil was placed over the L-DLPFC, positioned 5.5 cm anterior to the identified site of the motor cortex (Burke et al., 2019; Pascual-Leone et al., 1996). No additional normalization parameters were applied for RMT.

By using this standardized scalp-based measurement, stimulation site and coil positioning were consistently verified across treatment sessions. In line with established rTMS guidelines, the energy was delivered at 120% of RMT (Milev et al., 2016).

#### 2.3.2 Protocols

Both dTMS and iTBS protocols were delivered in accordance with Health Canada-approved parameters.

##### 2.3.2.1 The dTMS protocol

The Brainsway Deep TMS H1-coil delivered high frequency dTMS (HF-dTMS) over the L-DLPFC at 120% RMT (Kaster et al., 2019; Levkovitz et al., 2015; Tendler et al., 2016). The dTMS coil is capable of stimulating a broader field while penetrating deeper depths of up to 5 cm (Roth et al., 2007). In line with validated guidelines, treatment parameters were set at 18 Hz, 2 s trains and intertrain interval of 20 s, producing a total of 55 trains and 1980 pulses per session for the duration of 20.2 min (Kedzior et al., 2015; Levkovitz et al., 2015).

##### 2.3.2.2 The iTBS protocol

The Figure 8 coil was used to deliver stimulation at 120% RMT over the L-DLPFC (Blumberger et al., 2018; Holzer and Padberg, 2010; Kaster et al., 2019). Treatment parameters were set at triplet 50 Hz bursts, 2 s trains and intertrain interval of 8 s, producing a total of 20 trains and 600 pulses per session for the duration of just over 3 min, consistent with established guidelines (Blumberger et al., 2018; Burke et al., 2019). While the coil used for iTBS does not differ from standard rTMS, this protocol was used to provide an efficient alternative to the standard treatment course (Blumberger et al., 2018).

### 2.4 Clinical and demographic data analysis

IBM SPSS Version 27 (IBM Corporation, Armonk, New York, USA) was used for the statistical tests and graphic illustrations of the obtained data. The tests included a bivariate linear mixed model (LMM) featuring the factors of group, time, and group × time interaction effects of the variables of interest. For the clinical data, these variables were the changes in HDRS-17 and QIDS-16SR scores, presented as ∆HDRS-17 and ∆QIDS-16SR. For patient classification, remitters were defined as patients who achieved HDRS-17 score of <8 post-treatment, in line with established standards within the literature (Blumberger et al., 2018). To analyze clinical outcomes, the trajectories of ∆HDRS-17 and ∆QIDS-16SR were investigated for the MDD group across time. Additionally, the two group comparisons explored were dTMS vs. iTBS and remitter vs. non-remitter. Demographic data were analyzed at three comparison levels of MDD vs. HC, dTMS vs. iTBS, and remitter vs. non-remitter in order to confirm age-, sex-, and education-matched samples. Age was compared by conducting independent samples t-test across groups, while sex ratio and education level were compared by Fisher’s exact test and chi-square analysis, respectively. Missing data were accounted for by implementing the imputation method last observation carried forward (LOCF). Bonferroni correction was applied within each outcome domain (clinical, demographic, and cognitive assessments) to observe adjusted alpha levels (αadj) in order to control for possibility of type I errors due to multiple evaluations.

### 2.5 Cognitive assessment

#### 2.5.1 CANTAB

The Cambridge Neuropsychological Test Automated Battery (CANTAB; Cambridge Cognition, 2023) was used as a key instrument for assessing various cognitive domains including social–emotional cognition, executive function, memory, and attention. Delivered through a computerized platform, this well-established application has been validated in peer-reviewed publications investigating cognition in various neuropsychiatric conditions (Cambridge Cognition, 2023). In this study, the selected tasks were conducted in the following consecutive order: the Emotion Recognition Task (ERT) which assessed for the ability to identify six primary human emotions in the form of facial expressions; One Touch Stockings of Cambridge (OTS) as a derivation of the Tower of Hanoi which evaluated executive function; Verbal Recognition Memory (VRM) to measure verbal memory through the ability to process and retrieve verbal information; and lastly, the Rapid Visual Information Processing (RVP) task which evaluated the capacity for sustained attention. CANTAB cognitive assessment was 40 min in duration, completed at the time points of Week 0 (baseline), Week 2, Week 4, Week 6 (final visit), and Week 10 (follow-up). The most representative variables of the CANTAB tests were chosen with respect to the focus of the research question in order to explore patterns of association (details regarding CANTAB and explored outcome measures are listed in Supplementary materials).

#### 2.5.2 CANTAB score analysis

IBM SPSS Version 27 (IBM Corporation, Armonk, New York, USA) was used for the statistical tests and graphic illustrations. The tests comprised of a bivariate LMM featuring the factors of group, time, and group × time interaction effects of the variables of interest. Bivariate group analyses were performed at three levels of MDD vs. HC, dTMS vs. iTBS, and remitter vs. non-remitter. CANTAB provided age-, gender-, and education-matched normative data for select measures in RVP and OTS tasks. Z-scores were applied as they were derived from the CANTAB application which had incorporated the variables for age, gender, and education into its generated normative sample. To account for missing data, we implemented the imputation method LOCF (IBM Corp, 2020).

### 2.6 Attentional bias assessment

#### 2.6.1 Eye tracking paradigm

Eye tracking technology developed by Eizenman et al. (2003) at EL-MAR Incorporation (EL-MAR Inc. Toronto, Ontario, Canada) was used to investigate the reported presence of negative attentional bias in MDD participants relative to healthy controls, indicated by higher average glance duration on dysphoric images. The eye tracking apparatus was designed to track alternations in eye gaze positions as participants observed the slide deck, with each slide featuring four different images on the screen (Supplementary Figure 3).

#### 2.6.2 Visual stimuli characteristics

The slide deck consisted of 81 slides total, 32 test slides and 49 filler slides, with 15 min total presentation time (Eizenman et al., 2003). All featured images were selected from the publicly accessible and psychometrically validated normative databases, including the standardized facial expressions library of Karolinska Directed Emotional Faces (KDEF) (Lundqvist et al., 1998). The eye tracking task was conducted at the time points of Week 0 (baseline), Week 2, Week 4, Week 6 (final visit), and Week 10 (follow-up). KDEF slides featured the four facial expressions of happy, neutral, dysphoric, and disgust by the same actor to properly control for variables pertaining to visual properties of these images (refer to Supplementary materials for KDEF website). The role of the filler slides was to obfuscate the *a priori* chosen test slides while naturally acclimatizing the participants to the testing protocol.

#### 2.6.3 Testing procedures, VSB parameters, and analysis

Using the Visual Attention Scanning Technology software (VAST, 2015; EL-MAR Inc, 2015), eye gaze positions of participants were tracked through its binocular gaze estimation system (Guestrin and Eizenman, 2006) while the monitor presented the visual stimuli (Supplementary Figures 4–6). This test was administered in accordance with previous eye tracking studies (Chung, 2015; Eizenman et al., 2003). Eligible participants were required to have normal or corrected-to-normal vision, be seated at 60 cm distance from a 23-inch computer monitor, and view the 81-slide presentation with a steady gaze following a brief calibration procedure during which they were instructed to gaze and focus their eyes on 9 focal points on the screen (further details in Supplementary materials). Visual scanning behavior (VSB) variables of interest included relative fixation time (rFT), fixation frequency within image (FFWI), and average glance duration (AGD; measured in unit of ms) in order to explore the potential existence of attentional bias as a representation of the dysfunctional hot cognition in MDD. Data obtained from the 32 test slides were averaged to produce the values for average fixation time and average fixation frequency.

## 3 Results

### 3.1 Demographic features

The analyzed samples included the MDD group with a total of *N* = 24 patients who received rTMS (13 female, 11 male; mean age 41.13 ± SD 12.50) and the HC group with *N* = 33 participants (22 female, 11 male; mean age 44.12 ± SD 15.79). Chi-square and independent samples t-test analyses found the groups to be sex- [*X^2^*(1,57) = 0.916, *p* = 0.339], education- [*X^2^*(3,57) = 2.531, *p* = 0.470], and age-matched (t55 = −0.770, *p* = 0.180) (Table 1). *N* = 17 patients received dTMS (8 female, 9 male; mean age 42.18 ± SD 12.62) while iTBS was provided to *N* = 7 patients (5 female, 2 male; mean age 38.57 ± SD 12.82). These groups demonstrated comparable sex ratio of females and males (Fischer’s exact test, *p* = 0.386), were age-matched, (unpaired, two-tailed t-test, t22 = 0.63, *p* = 0.533) and had statistically similar intake in the number of psychiatric medications (unpaired, independent samples t-test, t22 = 0.07, *p* = 0.949). One-third of the MDD group (8 out of 24) were classified as remitters at the end of the acute rTMS course. There were no differences between remitters and non-remitters in age, sex, and concurrent psychiatric medication at baseline (*p* > 0.05 for all comparisons; Table 1).

**Table 1:** Demographic features overview.

Demographic measures	MDD (*N* = 24)		HC (*N* = 33)		Chi-square analysis	
	*N*	%	*N*	%		
Sex	13	54.17	22	66.67	X^2^ = 0.916	*p* = 0.339
Education (undergraduate)		65.22		60.60	X^2^ = 2.531	*p* = 0.470
Occupation (inactive)	18	75	3	9.68		
Relationship status (single)	11	45.83	14	43.75		
Completed treatment	22	91.67	n/a	n/a		

	MDD (Mean ± SD)	HC (Mean ± SD)	Independent samples t-test	
Age	41.13 ± 12.50	44.12 ± 15.79	T_55_ = 0.770	*p* = 0.180
Age of onset	26.26 ± 9.95	n/a		
Time to rTMS	18.31 ± 10.94	n/a		
% Lifetime affected	40.67 ± 17.43	n/a		
Number of psychiatric medications	2.46 ± 1.41	n/a		

Values for age and number of psychiatric medications are reported as mean plus or minus standard deviation.

Two MDD patients withdrew study consent after the Week 2 assessment due to a perceived lack of antidepressant efficacy from rTMS. Two HC participants also dropped out after completing the first visit at Week 0, due to one participant facing a medical issue and the other being lost to follow-up. Data from participants who dropped out of the study from both HC and MDD groups were included in the study analyses. None of the MDD patients were excluded from the study, however, four HC participants were excluded following the completion of the first study visit at Week 0 for various reasons including sudden discovery of meeting exclusion criteria, medical limitations, and complications. Data from these four excluded individuals were removed from the analyses as they were deemed ineligible. From the patient group, two individuals withdrew from the study before the Week 4 mark, one due to clinical nonresponse and the other as a result of experiencing anxiety about their perceived cognitive performance in the first study visit (for a visual illustration refer to the Supplementary Figure 2. CONSORT Flow Diagram in Supplementary materials).

### 3.2 Clinical outcomes

**Figure 1:**
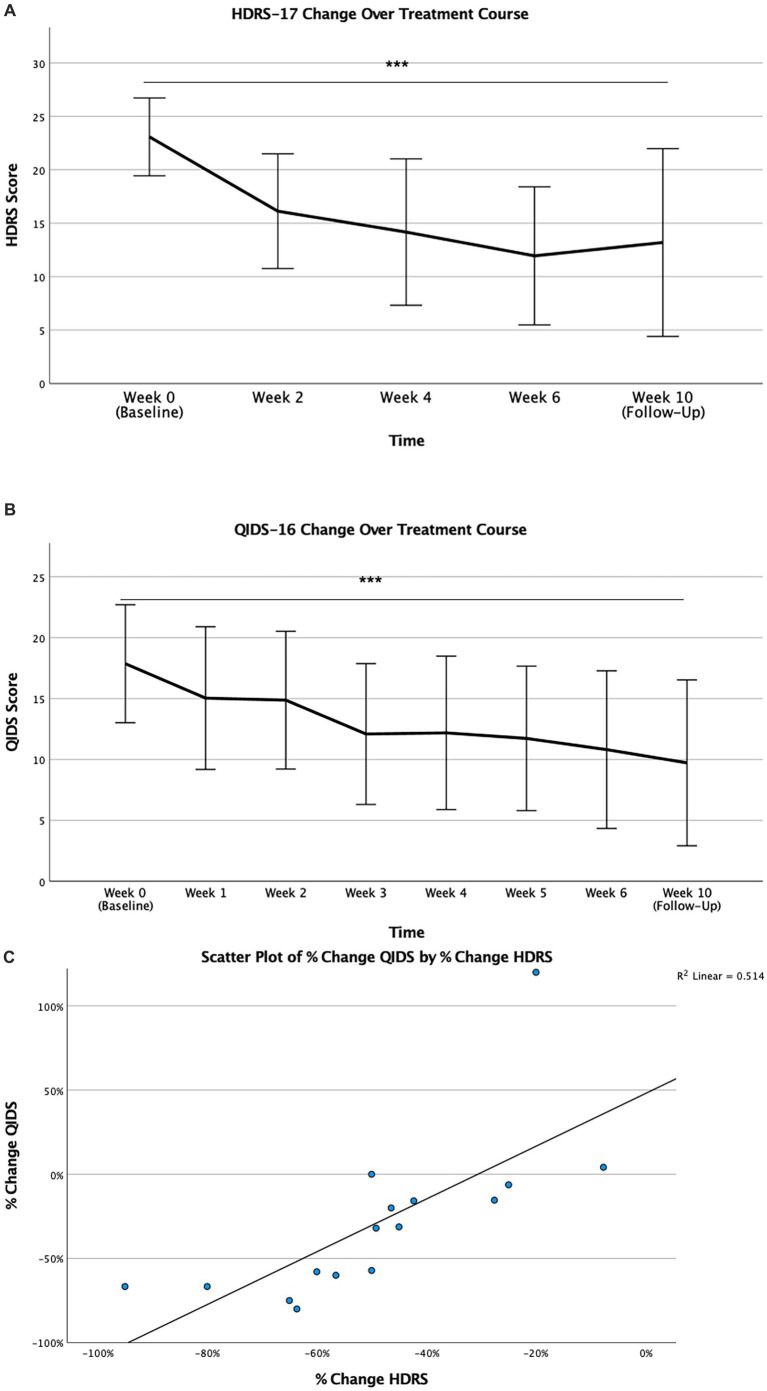
Depression severity during treatment course. Values are reported as mean HDRS-17 scores **(A)** and mean QIDS-16 scores **(B)** for all study time points. Error bars represent standard deviation of mean (*** for *p* < 0.001); Correlation of percent change of clinical improvement in depression severity as measured by QIDS-16 and HDRS-17 during treatment course **(C)**. Minus values indicate improvement and plus values represent worsening of clinical symptoms (r = 0.717, *p* = 0.002) (** for *p* = 0.002).

Clinical efficacy of rTMS was monitored by baseline depression severity scores pre-rTMS over time. Both clinician-rated HDRS-17 and self-report QIDS-16SR were used. For HDRS-17, analysis revealed a significant effect of time (*p* < 0.001), highlighting a significant decrease in HDRS-17 score at Week 2 and subsequent time points, suggesting an early and sustained antidepressant effect seen both during the acute treatment course and the Week 10 follow-up assessment. A similar early and sustained antidepressant effect was reported by patients on the QIDS-16SR, seen by Week 1 and maintained at Week 10 (Follow-Up). Correlation between objective HDRS-17 and the subjective QIDS-16SR reflecting changes in the degree of depressive symptomatology across treatment course was explored. Percent change of clinical improvement for QIDS and HDRS scores at final visit (Week 6) relative to baseline (Week 0) were found to be strongly correlated (r = 0.717, *p* = 0.003) (Table 2; Figure 1), indicating congruence between clinician and patient assessment of depressive symptoms. These analyses remained significant following Bonferroni correction (αadj <0.0033).

**Table 2:** Correlation of percent change of clinical improvement in depression severity as measured by QIDS-16 and HDRS-17 during treatment course.

Percentage of clinical improvement (%)	
Measure	% Change
QIDS-16	−32.01 ± 50.4 **
HDRS-17	−49.18 ± 22.3 **

Minus values indicate improvement and plus values represent worsening of clinical symptoms (r = 0.717, *p* = 0.002) (** for *p* = 0.002).

### 3.3 Cognitive outcomes

#### 3.3.1 Cold cognition

##### 3.3.1.1 Rapid visual information processing (RVP)

**Figure 2:**
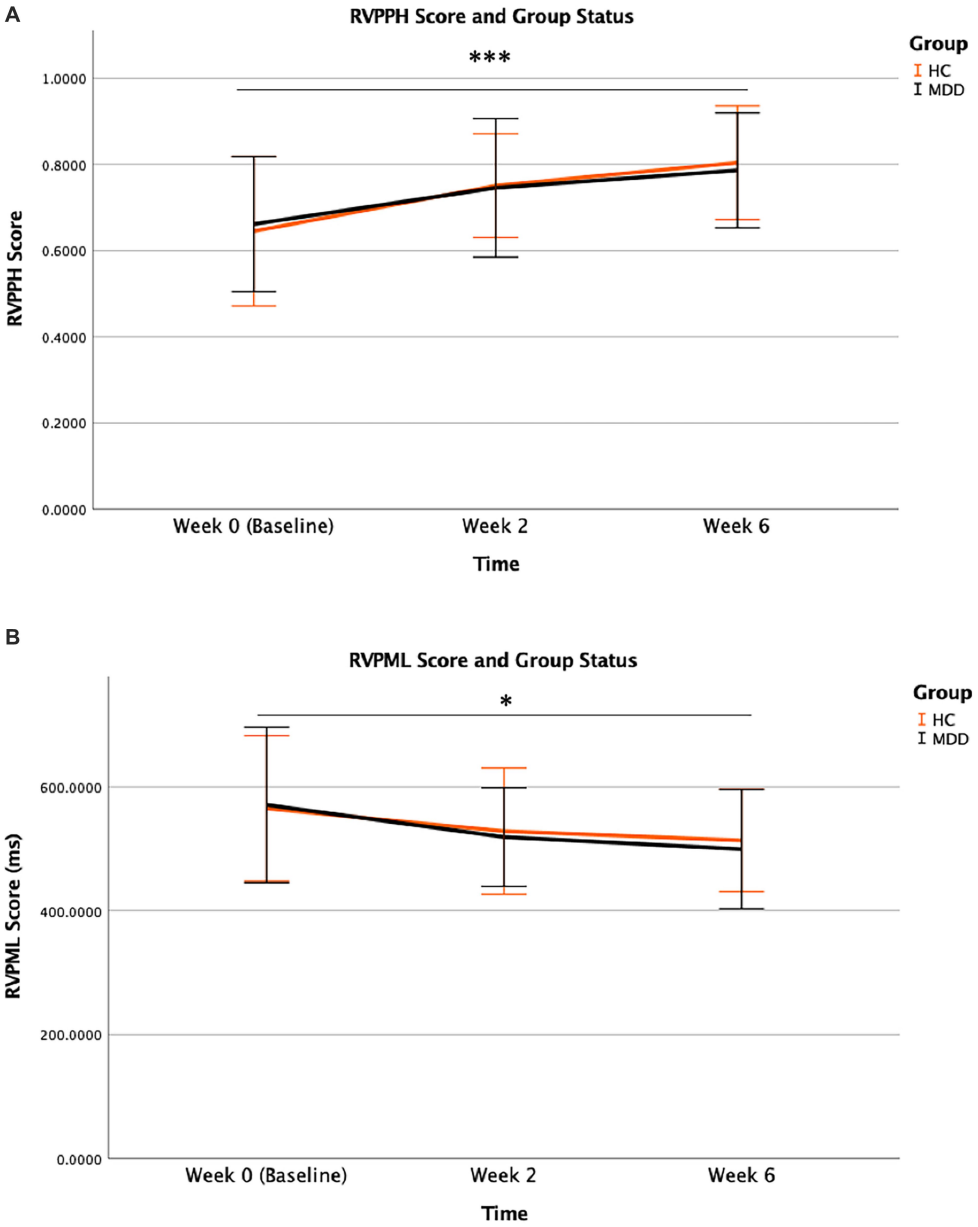
RVPPH **(A)** and RVPML **(B)** performance during treatment course according to remission status. Mean scores for all study time points from baseline (Week 0) to follow-up (Week 10) are illustrated for remitters and non-remitters. Error bars represent standard deviation of mean [*for *p* = 0.022, **for *p* = 0.001 **(A)**].

Improvements in sustained attention were observed over time in both MDD and HC groups, with no significant differences in performance among them. For RVPPH, the effect of group status [*F* (1,138) = 0.007, *p* = 0.935], and group × time interaction [*F* (2,138) = 0.154, *p* = 0.857] were not significant. However, the effect of time [*F* (2,138) = 10.864, p < 0.001] showed significance particularly following baseline (t = −2.580, *p* = 0.011), indicating that both MDD and HC participants improved significantly at Weeks 2 and 6. Similarly, group and group × time interaction effects were absent for RVPML and RVPA’ and only a significant time effect was observed for these two variables [*F* (2,138) = 4.428, *p* = 0.014 for RVPML, and *F* (2,138) = 10.565, *p* < 0.001] for RVPA’ (Figure 2). CANTAB database provided normative data for RVPA’ to which the MDD and HC groups in this study were compared (Table 3). For the outcome measure RVPA’, performance of MDD and HC groups matched that of CANTAB normative sample, illustrated by standardized scores at both baseline (Week 0) (t = 0.242, *p* = 0.810) and final visit (Week 6) (t = −0.550, *p* = 0.586).

**Table 3:** Standardized RVPA’ performance in reference to CANTAB normative data.

RVP A’ standardized score		
Group	Week 0 (Baseline)	Week 6
MDD	−0.04 ± 0.82	0.55 ± 0.93
HC	−0.09 ± 0.87	0.72 ± 0.91

Mean standardized RVP A’ scores (z-scores) in MDD and HC participants are reported as group mean plus or minus standard deviation. No value presented was significant.

**Figure 3:**
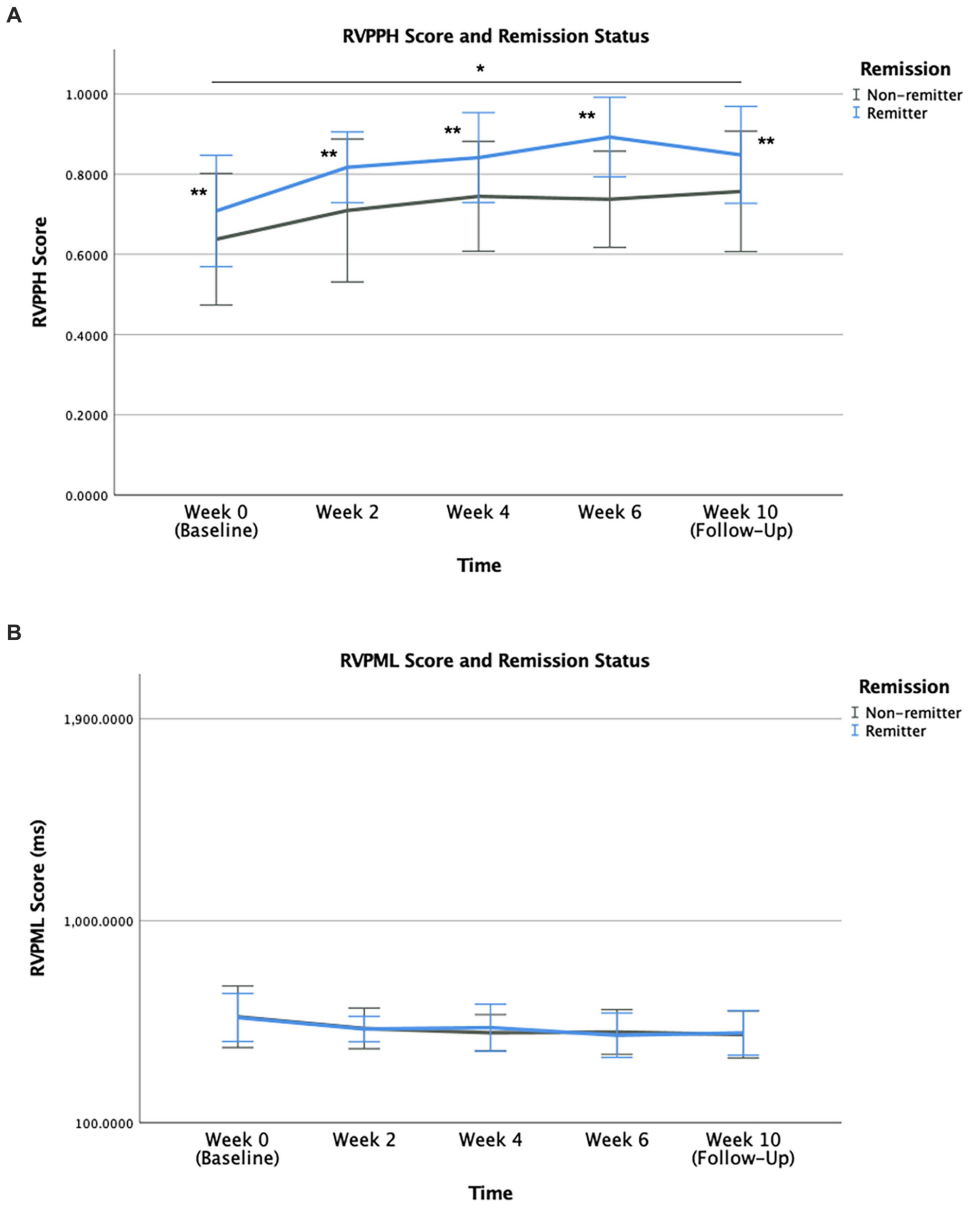
RVPPH **(A)** and RVPML **(B)** performance during treatment course according to group status. Mean scores for all study time points from baseline (Week 0) to final visit (Week 6) are illustrated for MDD and HC participants. Error bars represent standard deviation of mean (***, * for *p* < 0.001 and *p* = 0.014, respectively).

Remitters exhibited greater sensitivity toward target sequences and a superior ability for correctly responding to them. For RVPPH, remitters demonstrated superior performance compared to non-remitters across time (Figure 3) as they correctly responded to a greater number of target sequences. Specifically, group × time interaction [*F* (4,88) = 0.192, *p* = 0.942] was absent, while significant time [*F* (4,88) = 3.018, *p* = 0.022] and group [*F* (1,88) = 11.083, *p* = 0.001] effects were found. This group effect was sustained across all study time points. Similarly, for RVPA’, the superior performance of remitters relative to non-remitters across time was indicative of their greater sensitivity toward target sequence. Group × time interaction [*F* (4,88) = 0.176, *p* = 0.950] was absent, however, the effects of time [*F* (4,88) = 2.936, *p* = 0.025] and group [*F* (1,88) = 11.364, *p* = 0.001] were significant. This group effect was sustained across all study timepoints. For RVPML, the effects of time, group, and group × time interaction were absent (Figure 3). Superior performances of remitters in these select measures of the RVP task were independent from clinical outcome. No association was found between baseline RVPPH and clinical outcome as measured by the degree of change in HDRS-17 (% change of HDRS-17) from final visit (Week 6) relative to baseline (Week 0) (r = −0.025, *p* = 0.922). Baseline RVPA’ was also not associated with clinical outcome (r = −0.034, *p* = 0.893). The group effects for RVPPH and RVPA’ between remitters and non-remitters remained significant following Bonferroni correction (αadj < 0.0016).

##### 3.3.1.2 Verbal recognition memory (VRM)

**Figure 4:**
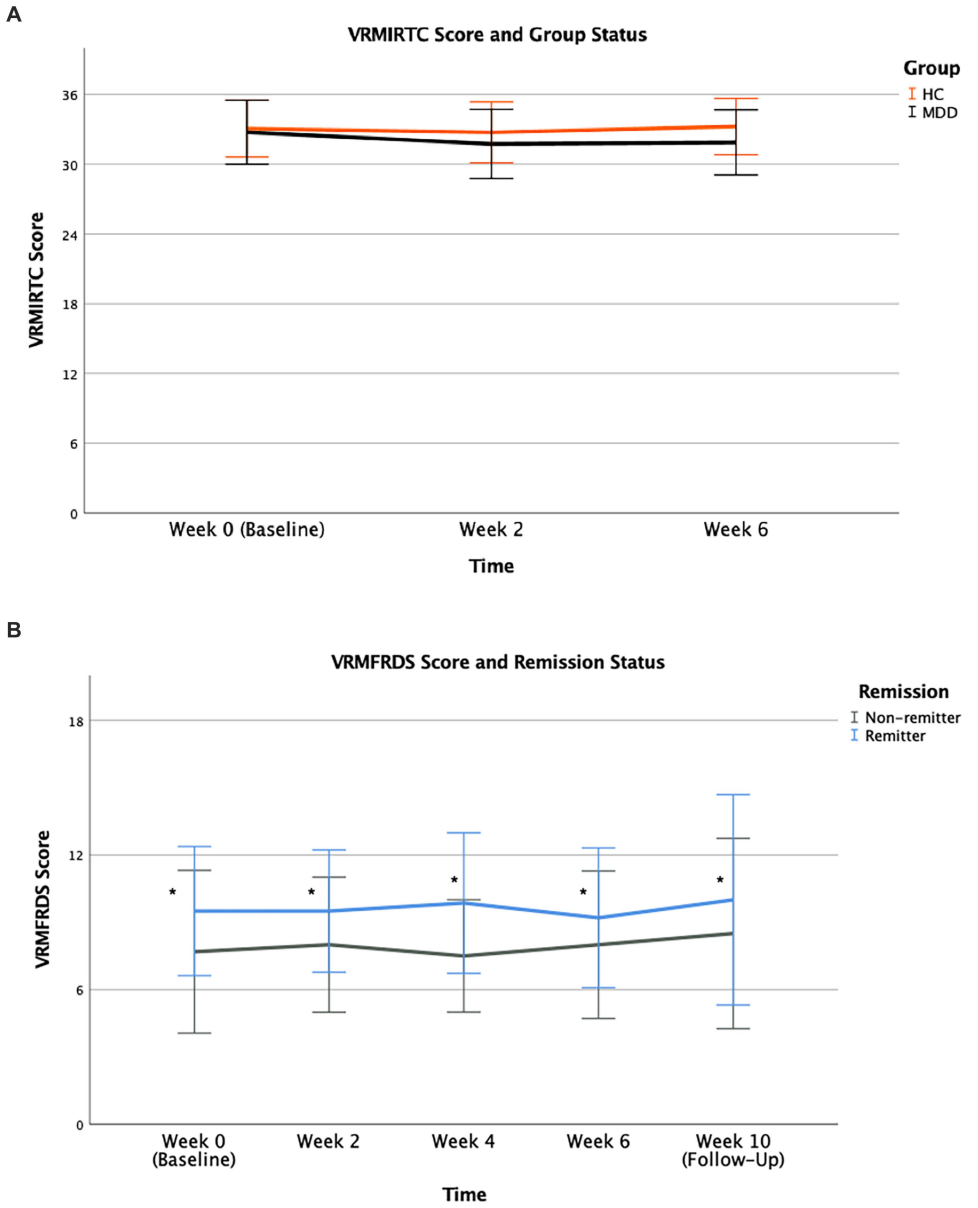
VRMIRTC **(A)** in MDD and HC, and VRMFRDS **(B)** in remitters and non-remitter groups, during treatment course. Mean scores for all study time points are illustrated along with error bars representing standard deviation of mean (* for *p* = 0.022).

Analysis for verbal recognition memory showed a near-significant difference in performance of both groups for the outcome measure VRMIRTC, as MDD patients demonstrated a trend toward inferior performance in immediate recognition of word choices compared to HC group across time (Figure 4). The effect of time [*F* (2,138) = 0.819, *p* = 0.443] and group × time interaction [*F* (2,138) = 0.475, *p* = 0.623] were not significant. The effect of group status [*F* (1,138) = 3.850, *p* = 0.052] was near-significant, as MDD group underperformed compared to HC group sustained across all study timepoints.

Remitters were found to have greater ability for free recall of distinct words compared to non-remitters. As measured by VRMFRDS, performance in free recall of distinct words was superior in remitters as they recalled a greater number of distinct words compared to non-remitters across time (Figure 4). The effect of time [*F* (4,88) = 0.093, *p* = 0.984] and group × time interaction [*F* (4,88) = 0.076, *p* = 0.989] were not significant. The significant group effect [*F* (1,88) = 5.415, *p* = 0.022] was sustained across all study timepoints. Superior performance of remitters in this outcome measure was independent from clinical outcome as no association was found between baseline VRMFRDS and clinical outcome, measured by the degree of change in HDRS-17 (% change of HDRS-17) from final visit (Week 6) relative to baseline (Week 0) (r = −0.037, *p* = 0.884).

##### 3.3.1.3 Emotion recognition task (ERT)

**Figure 5:**
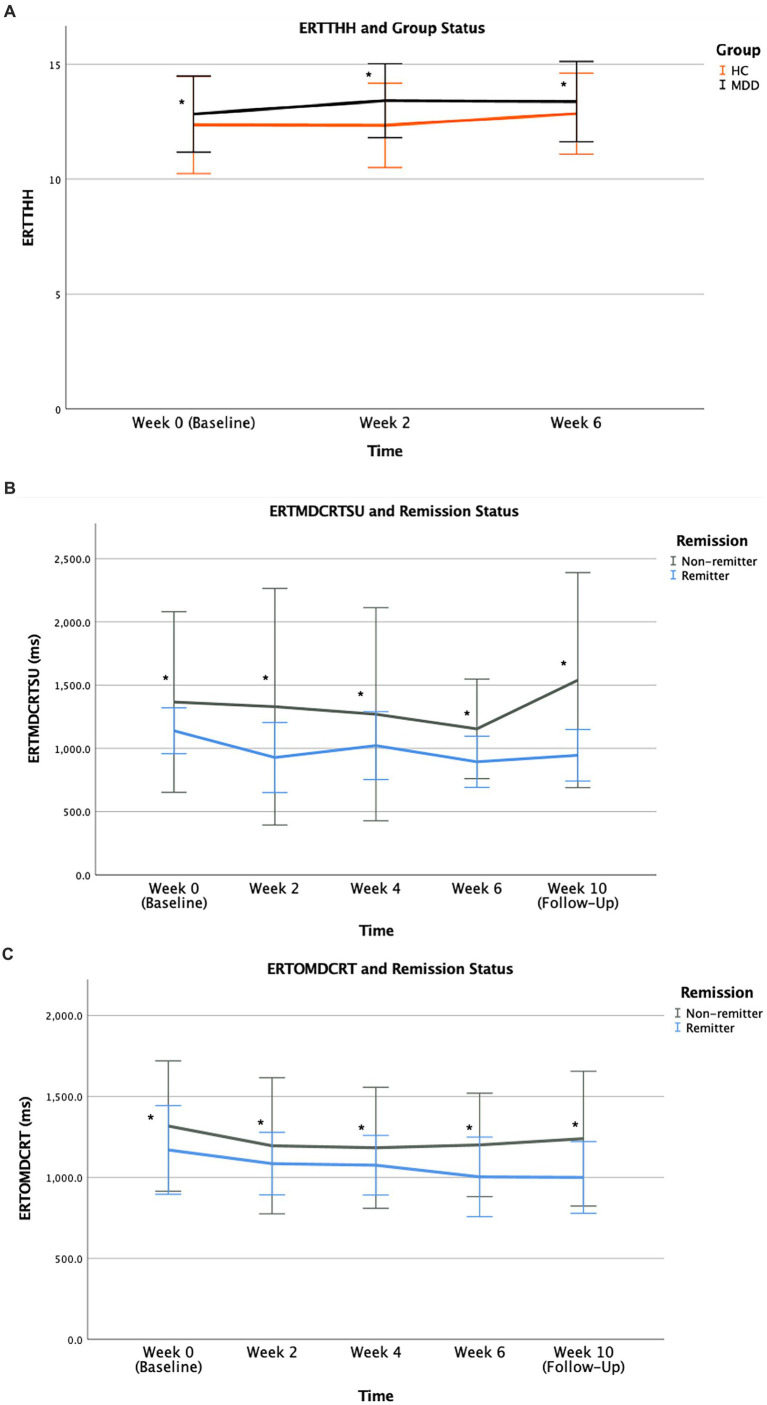
ERTTHH **(A)** performance during treatment course according to group status. Mean scores for all study time points from baseline (Week 0) to final visit (Week 6) are illustrated for MDD and HC participants. ERTMDCRTSU **(B)** and ERTOMDCRT **(C)** performance (unit of ms) during treatment course according to remission status. Mean scores for all study time points from baseline (Week 0) to follow-up (Week 10) are illustrated for remitters and non-remitters. Error bars represent standard deviation of mean [* for *p* = 0.029 **(A)**, * for *p* = 0.019 **(B)**, * for *p* = 0.038 **(C)**].

MDD patients outperformed the HC group in correctly identifying emotions. For total correct selections of individual emotions, relative to HC participants, the MDD group showed greater ability in correctly identifying and selecting for the emotion happiness (ERTTHH) across all assessed trials over the direction of treatment course (Figure 5). The effect of time [*F* (2,137) = 0.895, *p* = 0.411] and group × time interaction [*F* (2,137) = 0.410, *p* = 0.665] were not significant while the group effect [*F* (1,137) = 4.854, *p* = 0.029] was statistically significant. For other emotions of sadness, anger, and fear, MDD group also had superior performance in this outcome measure. However, both HC and MDD groups were comparable in identifying the emotions of surprise and disgust.

Remitters had faster emotion recognition as they exhibited shorter latency of correct selections for emotions. For median latency of correct selections of individual emotions, specifically for the emotion surprise (ERTMDCRTSU), remitters performed better as they were capable of correctly identifying the emotion surprise faster with a shorter reaction time relative to non-remitters across all study visits (Figure 5). The effect of time [*F* (4,88) = 0.315, *p* = 0.867] and group × time interaction [*F* (4,88) = 0.200, *p* = 0.938] were not significant. However, the group effect [*F* (1,88) = 5.740, *p* = 0.019] was significant, highlighting remitters’ superior performance sustained across all study timepoints. For overall median latency of correct selection for all emotions (ERTOMDCRT), remitters’ performance trajectory was notably different from non-remitters, such that remitters had superior performance with faster correct identification and selection of all emotions overall with a shorter reaction time compared to non-remitters (Figure 5). This finding was supported by the significant group effect [*F* (1,88) = 4.456, *p* = 0.038] sustained across all study timepoints while the effect of time [*F* (4,88) = 0.496, *p* = 0.739] and group × time interaction [*F* (4,88) = 0.102, *p* = 0.981] were not significant. Both baseline ERTMDCRTSU and baseline ERTOMDCRT were not associated with clinical outcome, measured by degree of change in HDRS-17 (% change of HDRS-17) from final visit (Week 6) relative to baseline (Week 0) (r < 0.001, *p* = 0.999 and r = −0.065, *p* = 0.799, respectively), suggesting these outcome measures in emotion recognition may function as stable factors associated with remission.

##### 3.3.1.4 One touch stockings of Cambridge (OTS)

**Figure 6:**
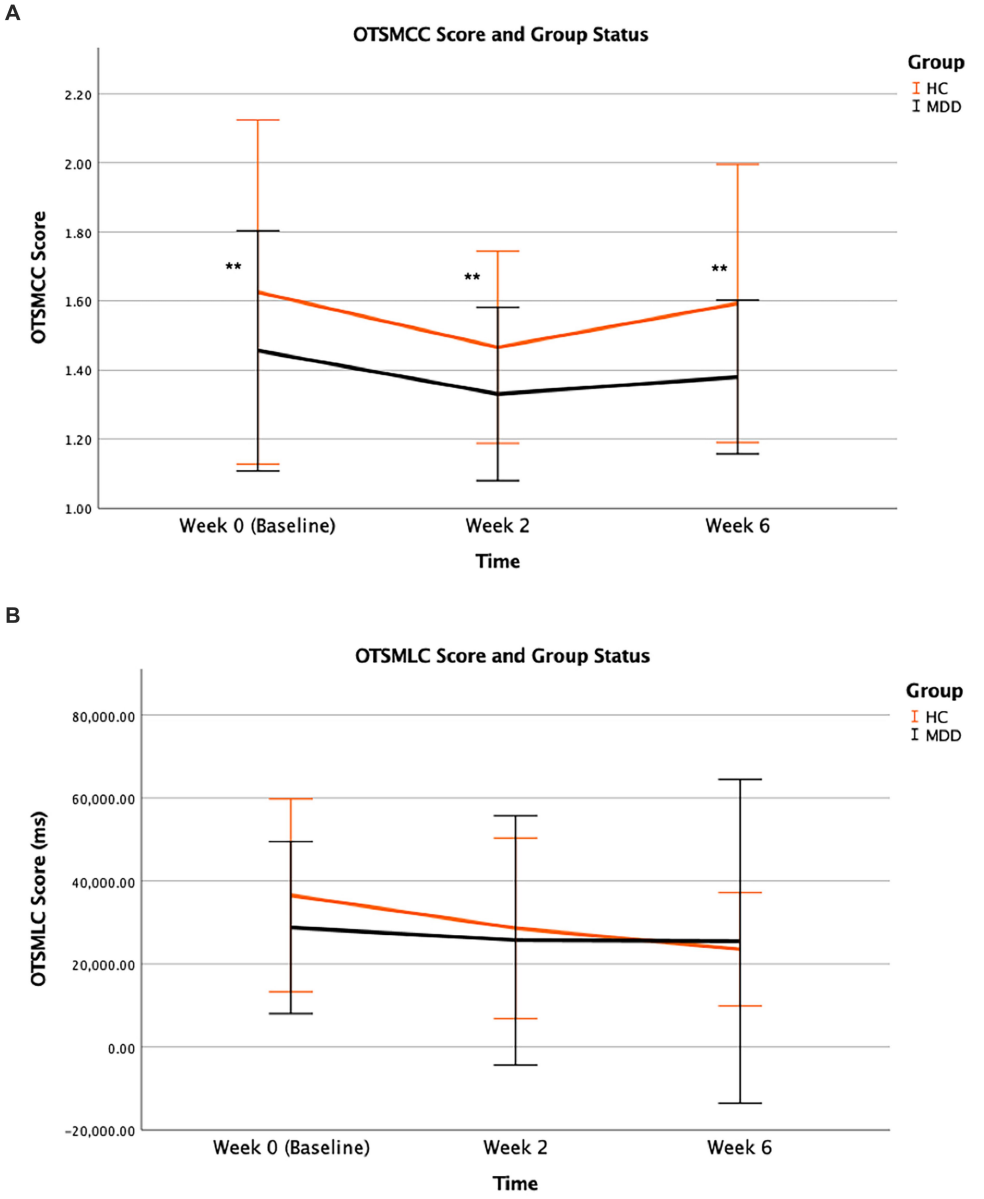
OTSMCC **(A)** performance and OTSMLC **(B)** scores (unit of ms) during treatment course according to group status. Mean scores for all study time points from baseline (Week 0) to final visit (Week 6) are illustrated for MDD and HC participants. Error bars represent standard deviation of mean (** for *p* = 0.006).

HC participants underperformed in some outcome measures of executive function relative to MDD group, while both groups had similar latency to correct response. For OTSMCC, HC group demonstrated underperformance compared to MDD patients sustained across all study time points as supported by the significant group effect (*p* = 0.006), while the effect of time (*p* = 0.128) and group × time interaction (*p* = 0.884) were found to be not significant (Figure 6). However, mean latency to correct choice as measured by OTSMLC was comparable in both groups, with non-significant effects of time (*p* = 0.280), group status (*p* = 0.498), and group × time interaction (*p* = 0.662) (Figure 6). With regards to problems solved on first choice, OTSPSFC, HC participants underperformed compared to MDD patients (group effect of *p* = 0.005). Both groups were assessed relative to the normative data provided by CANTAB. HC participants were also found to have underperformed compared to CANTAB’s normative sample, evidenced by lower standardized scores at both baseline (Week 0) (t = 0.899, *p* = 0.373) and significantly at the final visit (Week 6) (t = 2.347, *p* = 0.025) (Table 4).

**Table 4:** Standardized OTSPSFC scores in reference to CANTAB normative data.

OTSPSFC standardized score		
Group	Week 0 (Baseline)	Week 6 *
MDD	0.07 ± 1.03	0.33 ± 0.66 *
HC	−0.18 ± 1.07	−0.26 ± 0.82 *

Mean values are reported as z-scores indicating group mean plus or minus standard deviation. Values at Week 6 were significant (* for *p* = 0.025).

Executive function performance of remitters and non-remitters were similar for all explored outcome measures of the OTS task. No statistically significant effects were found for these measures on the basis of remission status.

#### 3.3.2 Hot cognition

**Figure 7:**
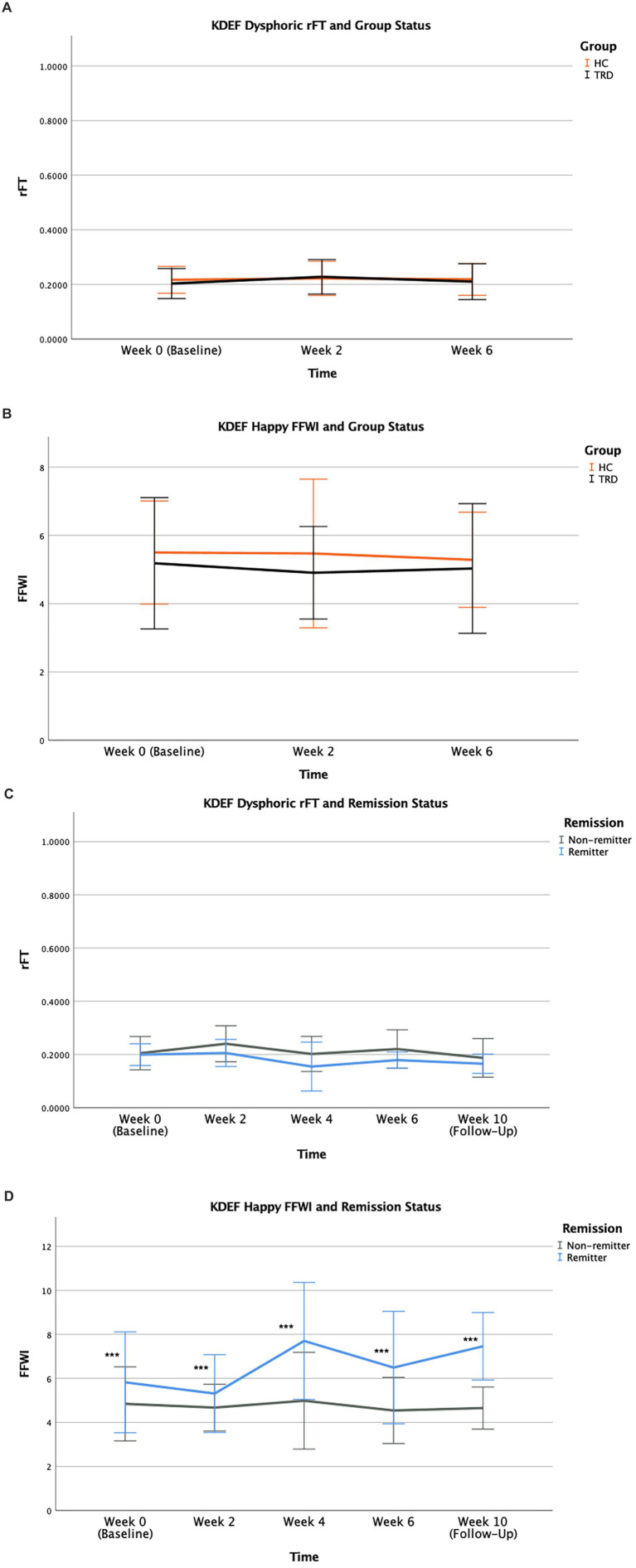
KDEF Dysphoric rFT in MDD vs. HC **(A)** and KDEF Happy FFWI in MDD vs. HC **(B)**; KDEF Dysphoric rFT in remitter vs. non-remitter **(C)** and KDEF Happy FFWI in remitter vs. non-remitter **(D)** for all study timepoints from baseline (Week 0) to follow-up (Week 10). Error bars represent standard deviation of mean (*** for *p* < 0.001).

Negative attentional bias was absent in the MDD group. Both groups demonstrated similar VSB toward neutral images. Positive attentional bias was also absent in healthy controls. With regards to VSB toward dysphoric faces, MDD and HC groups were comparable, supported by non-significant findings for rFT [*F* (1,125) = 0.262, *p* = 0.610], FFWI [*F* (1,125) = 0.159, *p* = 0.691], and AGD [*F* (1,125) = 0.021, *p* = 0.886]. The effect of time and group × time interaction were also not significant. Therefore, we found an absence of attentional bias toward dysphoric images in MDD (Figure 7). No significant group effect was observed for the outcome variables pertaining to VSB toward neutral faces between MDD and HC groups, supported by the analyses for rFT [*F* (1,125) = 0.060, *p* = 0.808], FFWI [*F* (1,125) = 0.333, *p* = 0.565], and AGD [*F* (1,125) = 0.219, *p* = 0.641]. The effect of time and group × time interaction were also found to be not significant. The parameters for VSB for disgust faces, namely the rFT and AGD, were comparable in MDD and HC participants [*F* (1,121) = 1.947, *p* = 0.166 for rFT; *F* (1,121) = 2.190, *p* = 0.142 for AGD]. However, for FFWI, there was a significant group × time interaction (*p* = 0.050) following baseline (Week 0) with HC having lesser FFWI compared to MDD which was shown to increase over the course the study. For protocol type, mean FFWI for disgust faces was significantly higher in the iTBS group relative to those receiving dTMS [*F* (1,74) = 6.823, *p* = 0.011] sustained across the study timeline from baseline (Week 0) to follow-up (Week 10). Rather than a therapeutic effect, this finding can be attributed to a potential marked random discrepancy at baseline between the groups and a regression to the mean. With respect to happy faces, the MDD and HC groups did not differ in VSB parameters of rFT [*F* (1,121) = 1.346, *p* = 0.248], FFWI [*F* (1,121) = 2.480, *p* = 0.118], and AGD [*F* (1,121) = 0.000, *p* = 0.993] (Figure 7), suggesting the absence of positivity bias in healthy controls.

**Figure 8:**
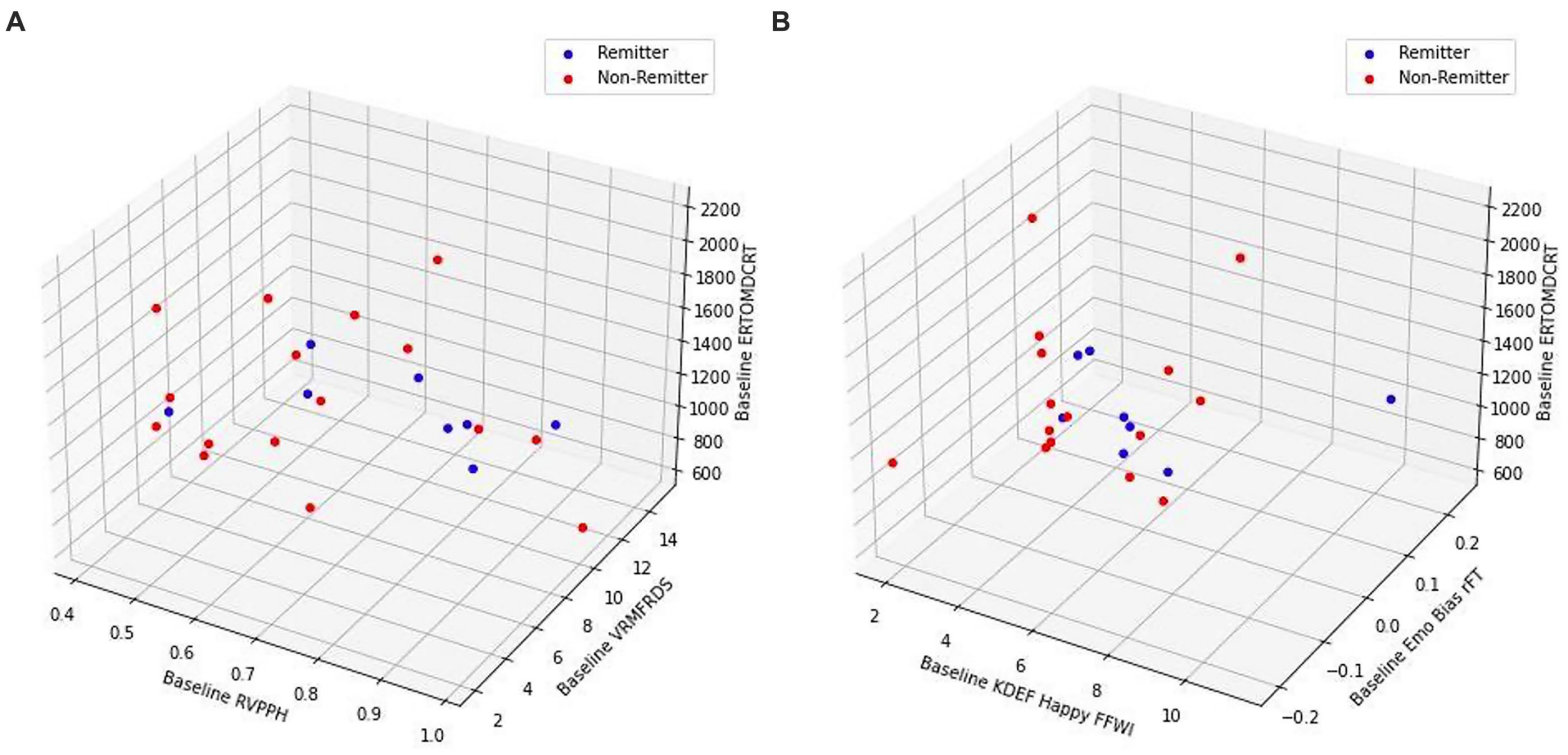
Three-dimensional visualizations of: cold cognition baseline phenotypic factors **(A)**, hot and cold cognition baseline phenotypic factors **(B)** distinguishing MDD patients based on remission status.

Negative attentional bias was absent in both remitters and non-remitters, and both groups had similar VSB toward neutral and disgust images. Frequency of fixation on happy images was distinctly higher in remitters. No significant effect was found with for VSB parameters in dysphoric faces when comparing remitters and non-remitters, observed for rFT [*F* (1,73) = 3.654, *p* = 0.060], FFWI [*F* (1,73) = 0.001, *p* = 0.973], and AGD [*F* (1,73) = 3.135, *p* = 0.081] (Figure 7). Both groups also displayed similar VSB toward neutral faces, supported by the absence of significant group effect for the parameters of rFT [*F* (1,73) = 0.000014, *p* = 0.997], FFWI [*F* (1,73) = 0.514, *p* = 0.476], and AGD [*F* (1,73) = 0.000015, *p* = 0.997]. Similarly, no significant group effect was found for VSB toward disgust faces for rFT [*F* (1,73) = 2.906, *p* = 0.093], FFWI [*F* (1,73) = 0.139, *p* = 0.710], and AGD [*F* (1,73) = 2.832, *p* = 0.097], suggesting the absence of attentional bias toward disgust images in both groups. Regarding happy faces, remitters and non-remitters demonstrated comparable VSB for rFT [*F* (1,73) = 2.408, *p* = 0.125] and AGD [*F* (1,73) = 0.145, *p* = 0.704]. However, a significant group effect was found for FFWI [*F* (1,73) = 17.031, *p* < 0.001] surviving Bonferroni correction (αadj<0.0018), with remitters having a greater FFWI on happy faces relative to non-remitters sustained across all study visits (Figure 7).

## 4 Discussion

This study had a 54% response rate and 33% remission rate for rTMS, aligned with empirical outcomes in the extant literature for response and remission rates of dTMS (47% and 42%, respectively) (Levkovitz et al., 2009) and iTBS protocols (49% and 32%, respectively) (Blumberger et al., 2018). Both dTMS and iTBS protocols had comparable clinical improvement trajectory and their clinical efficacy, as established in the literature, was further supported by the objective HDRS-17 and self-report QIDS-16SR clinical assessments. Another key finding was the association between the subjective impression of clinical response – evaluated by QIDS-16SR – and the objective evaluation of a mental healthcare professional – assessed by HDRS-17 – suggesting concordance between physician and patient reports. No minor or serious adverse events were reported and there was no worsening of performance across the assessed hot and cold cognitive domains. Therefore, in conjunction with the observed clinical effect of rTMS in our patient sample, this study further validates the established tolerability and safety of dTMS (Harel et al., 2011; Harel et al., 2014; Levkovitz et al., 2009) and iTBS protocols (Holzer and Padberg, 2010; Milev et al., 2016; Serafini et al., 2015).

In evaluating cold cognition, significant time effect was found in MDD and HC groups for all variables measuring sustained attention, reflecting substantial improvement over time. Regarding the CANTAB normative data for RVPA’, our findings highlighted the statistically similar performance of HC and MDD participants to the CANTAB normative sample, showcasing improvement by the final visit (Week 6). Overall, performance trajectories of RVPPH, RVPML, and RVPA’ were similar in MDD and HC participants, with both groups demonstrating significant improvement in sustained attention compared to baseline, suggesting the role of practice effects in this observation (Hopman et al., 2021). For verbal memory performance, a near-significant group effect was observed for total correct immediate word recognition (VRMIRTC) in the VRM task, with MDD patients underperforming consistently compared to HC group (*p* = 0.052) across all visits, indicating depressed patients correctly recognized and rejected fewer target words and distractor words, respectively. This underperformance further validates and is supported by the evidence in literature reporting deficiency in verbal memory and working memory within this patient population (Gupta et al., 2013; McIntyre et al., 2013; Pu et al., 2018; Woo et al., 2016). Moreover, the absence of a time effect for this variable suggested no improvement in verbal memory in patients following rTMS.

Exploring emotion recognition with the ERT task served as a measure of emotional bias from a cold cognition perspective and evaluated the conscious recognition of emotion-laden facial expressions. MDD patients outperformed healthy controls in total correct hits, involving both identification and selection, of facial expressions of happiness, sadness, fear, and anger. This finding reinforces past research reporting greater accuracy of sad facial expressions in individuals with depression and is suggested to be explained by the recognized mood-congruent sadness bias in depression (Eizenman et al., 2003; Kan et al., 2004; Leyman et al., 2011). For emotions of surprise and disgust, both groups performed similarly possibly due to the greater complexity of these expressions. While some report similar recognition of disgust in both healthy and depressed individuals (Bourke et al., 2010; Kan et al., 2004), the literature presents mixed findings, with other studies highlighting greater difficulty for MDD patients in accurately identifying happy, disgust, and neutral expressions (Krause et al., 2021). Overall, greater emotional awareness in individuals with depression may explain MDD patients’ outperformance in this task and in their ability for correct emotion recognition; a phenomenon that has been recognized in empirical findings noting that individuals with MDD who were unsuccessful in their recovery possess a greater degree of attention to emotion relative to those who recovered and never-depressed individuals (Thompson et al., 2013).

Executive function was evaluated through the OTS task where time effect for its investigated measures was absent. Relative to the MDD group, the HC participants underperformed consistently in outcome measures pertaining to mean choices to correct (OTSMCC) and total number of problems solved on first choice (OTSPSFC), while the mean latency to correct choice (OTSMLC) was comparable in both groups. This finding indicates that healthy individuals had the tendency to make more choices to achieve the correct answer and were able to solve fewer problems on first choice. As evidenced by negative standardized scores, healthy participants also underperformed compared to CANTAB normative data for OTSPSFC. These observations are in contrast with the impaired higher-order executive functioning and problem-solving abilities found in MDD relative to healthy controls (McIntyre et al., 2013; Pu et al., 2018; Woo et al., 2016). According to the anecdotal reports of the study administrator, healthy participants exhibited less drive and motivation compared to MDD patients in completing study tasks which may limit the potential generalizability of these findings. To conclude, rTMS was found to not induce significant changes in cold cognition and both protocols of dTMS and iTBS had comparable outcomes. This observation does not provide support for the model that rTMS exerts its influence on cold cognition through top-down cognitive control (Roiser and Sahakian, 2013). While some findings corroborate with this framework (Rostami et al., 2022), our observations suggest the mechanism underlying mood improvement as a result of rTMS may be distinct from any potential influence on cognition. We found improvements in cognitive performance over time in the domains of sustained attention and emotion recognition, however, since they were observed in both depressed and healthy participants, the changes are likely attributed to practice effects rather than salutary effects of rTMS on individuals with major depression.

In assessing hot cognition, for happy images, analyses at both levels of MDD vs. HC and dTMS vs. iTBS reported no difference among groups in all outcome measures. These results are contrary to past findings suggesting the existence of a significant positive attentional bias in healthy relative to depressed individuals (Roiser et al., 2012: Suslow et al., 2020). We also found negative attentional bias toward dysphoric images to be absent in MDD patients compared to HC individuals which does not align with other research highlighting the existence of negative attentional bias in depression (Eizenman et al., 2003; Roiser et al., 2012). Moreover, while other studies report rTMS to be associated with the alleviation of negativity bias in responders (Leyman et al., 2011), our study diverged from this observation, proposing the possibility that implicit attentional preferences toward dysphoric visual stimuli are not influenced by the mechanism of rTMS. Additionally, MDD patients demonstrated significantly greater fixation frequency within disgust images compared to healthy controls, indicated by a group × time interaction following baseline which was subsequently increased over the study course. This finding may corroborate with the literature stating individuals with depression experience greater difficulty in recognizing disgust (Krause et al., 2021) as the more frequent fixations within the image can be interpreted to reflect a pattern of passive and implicit processing that requires more time to analyze the image. No significant differences in attentional measures for neutral faces were found between depressed and healthy participants, remitters and non-remitters, and both protocols. Both dTMS and iTBS protocols were comparable in their attentional outcomes. Given the absent time effect, it is possible that rTMS has no influence on neutral and emotion-independent processing.

By exploring the association between cold cognition and remission, we identified a baseline remitter cognitive phenotype. Concerning sustained attention, while both groups demonstrated improvement in performance across time highlighted by significant time effect, remitters exhibited significantly superior performance at all time points relative to non-remitters in select measures of the RVP task, specifically probability of hit (RVPPH) and detection sensitivity to target sequence (RVP A’). These measures were found to be independent from clinical outcome, therefore, greater propensity for sustained attention and present awareness might be characteristic features of remitters and represent stable factors associated with clinical remission. This finding is in parallel with results by Hopman et al. (2021) where they also reported a differential performance trajectory between responders and non-responders regarding detection sensitivity to target sequence, with responders showcasing greater performance and magnitude of improvement. Moreover, the two groups did not differ in mean latency of correct response to target sequence, demonstrating equivalent speed in this domain. This observation can be explained by considering this parameter as a measure for reaction time and response speed rather than sustained attention. Evidence in the literature corroborates with our finding as remitters have exhibited faster reaction times than non-remitters in task switching, exhibiting this prominent difference pre-rTMS (Abo Aoun et al., 2019). Regarding verbal memory, remitters demonstrated a significantly superior ability to freely recall distinct words across all study time points. Therefore, we suggest that free word recall may represent another component of the remitter cognitive phenotype and serve as a stable factor associated with remission. For emotion recognition, remitters performed significantly greater at all time points with respect to median latency of correct reaction time for identification of all facial expressions (ERTOMDCRT measure), demonstrating faster overall identification of facial expressions with diverse emotional valence compared to non-remitters. This result was particularly pronounced for the expression of surprise, which was identified by remitters with a significantly shorter latency. Since ERTOMDCRT was found to not be correlated with clinical outcome as measured by HDRS-17, it is suggested to be independent from mood and may signify a trait feature of the cognitive phenotype of remitters. Figure 8 showcases the three-dimensional clustering of both groups with non-remitters positioned at a lower baseline RVPPH and higher ERTOMDCRT, representing compromised sustained attention and reduced speed of correct emotion recognition. Conversely, the cognitive phenotype for remitters suggests their faster emotion recognition, robust sustained attention, and notable verbal memory performance at baseline. Both remitters and non-remitters had comparable executive function performance in all outcome measures of the OTS task. Our results indicated that identified cold cognitive phenotype features pertaining to sustained attention, emotion recognition, and verbal memory were not significantly associated with clinical outcome, suggesting the possibility that the cold cognition elements of the remitter cognitive phenotype represent trait, rather than state, markers for patients with depression. In summary, our results validate the clinical efficacy and cognitive safety of rTMS and propose potentially distinct mechanisms underlying mood improvement and cognition. Moreover, our findings corroborate with the literature in highlighting the predictive value and clinical utility of classification of treatment response and identification of treatment outcome biomarkers for optimizing personalized care (Ebrahimzadeh et al., 2021; Ebrahimzadeh et al., 2023; Ebrahimzadeh et al., 2024; Nikravan and Ebrahimzadeh, 2021).

The present study demonstrates several notable strengths. Firstly, the inclusion of an age-, sex-, and education-matched healthy control group was a pivotal addition to the study design, especially given its absence in studies within this field, as it allows for the delineation of rTMS-induced effects from practice effects (Hopman et al., 2021). Given that long-term studies are limited in the literature (Asgarinejad et al., 2024), this study contributes to the field in exploring both acute and follow-up phases of treatment with its longitudinal design and a 91% treatment completion rate, while evaluating the potential differential effects of two different protocols on cognition. Linear mixed model (LMM) was chosen as the primary statistical technique for its strength in effectively accounting for missing values and the tests were conducted at three comparison levels (MDD vs. HC, dTMS vs. iTBS, and remitter vs. non-remitter), offering a highly detailed approach to our investigation. Additionally, the broad selection of cognitive outcome measures was utilized to investigate both hot and cold cognition simultaneously. Several cold and hot cognitive measures spanning the domains of sustained attention, verbal memory, emotion recognition, executive function, and visual scanning behavior did not show statistically significant results, inferring no difference between depressed and healthy control groups and an absence of rTMS-induced effect. As highlighted earlier, some outcomes may be attributed to mere practice effects. Cognitive outcome measures may also be highly correlated, therefore, there is a greater possibility for multicollinearity and type I errors which can be mitigated by minimizing the level of significance to *p* < 0.01. Other limitations include the small total sample size of *N* = 59 (MDD group *N* = 24, HC group *N* = 35) which compromises the findings’ statistical power and is a frequently reported limitation in the literature (Hopman et al., 2021), as well as the absence of objective measures to account for participant motivation.

Future research will greatly benefit from larger sample sizes as it will enhance the statistical power of results and adopting longitudinal design. Although we strengthened the research design by including a healthy control group, non-significant results may be due to potentially underpowered findings as a result of limited sample size. Inclusion of a healthy control group highlighted some differences in cognition among MDD patients and the control group, however, larger sample sizes in future studies may facilitate the detection of subtle differences which may aid in elucidating group × time interaction effects. It will also be advantageous to implement neuroimaging techniques to investigate the potential mechanisms including functional connectivity changes (Asgarinejad et al., 2024) underlying the reported modest cognitive improvement associated with rTMS in order to distinguish whether they signify trait markers, are a result of inflated type II errors due to absent cognitive deficits, or whether they reflect difficulty in identifying existing cognitive deficits due to their underlying neurological compensatory mechanisms (Hopman et al., 2021). To progress clinical efforts in research optimization, different rTMS treatment parameters can be investigated including various target sites and coil types, stimulation frequency and intensity, as well treatment duration (Fu et al., 2025), while also using neuronavigation-based adjustments for rTMS delivery to control for anatomical parameters such as scalp-to-cortex distance and account for degree of stimulation (Stokes et al., 2005). Moreover, incorporating well-established instruments for evaluating baseline general intelligence, such as the Wechsler Adult Intelligence Scale – Fourth Edition (WAIS-IV), as well as objective assessments for motivation and drive such as Effort Expenditure for Rewards Task (EEfRT), will benefit future studies as it will clarify the potential role of general intelligence and motivation as confounding factors in cognitive performance (Treadway et al., 2009; Treadway et al., 2012; Wechsler, 2014). To preserve consistency among studies, we recommend the incorporation of universally accessible assessments for neurocognitive performance (Serafini et al., 2015) - such as CANTAB - as they promote the synthesis and transability of findings across various studies with a greater cohesion. Ultimately, prospective studies are encouraged to investigate the impact of potential confounding variables - such as age, sex, and education level – at the intersection of cognition and clinical outcomes, and explore other factors associated with our study’s novel cognitive phenotype of remitters to further characterize the features of rTMS remitters for the advancement of optimized and personalized care.

## 5 Research resource identifiers (RRIDs)

The RRIDs for tools and resources used in this study are as follows:

HDRS-17 – Hamilton Rating Scale for Depression (RRID: SCR_003686).

Cold Cognition was assessed using CANTAB from Cambridge Cognition (RRID: SCR_003001).

## Data Availability

The raw data supporting the conclusions of this article will be made available by the authors, without undue reservation.
