# Supplementary material for: Identification of cognitive predictors of remission in depression following limited effect of repetitive transcranial magnetic stimulation on hot and cold cognitive systems
Source: Front Hum Neurosci. 2025 Dec 17;19:1696560. doi: 10.3389/fnhum.2025.1696560 (PMC12753519; doi:10.3389/fnhum.2025.1696560)
Supplement: Supplementary file 1 [file Data_Sheet_1.docx]

*Supplementary Materials for the Manuscript “Identification of Cognitive Predictors of Remission in Depression Following Limited Effect of Repetitive Transcranial Magnetic Stimulation on Hot and Cold Cognitive Systems”*

# Supplementary Data and Resources

## Cold Cognition

### CANTAB – Description of Outcome Measures

CANTAB task was 40 minutes long, with a breakdown of ERT (9 min), OTS (10 min), VRM (10 min), and RVP (9 min) tests.

Explored outcome measures for each task include the following:

RVP: The outcome measures explored in this task to evaluate sustained attention included probability of hit (correct hits ÷ total number of sequences) (RVPPH), mean response latency on trials where participant correctly responded (RVPML), and signal sensitivity towards detection of target sequence (RVPA’).

VRM: The outcome measures investigated for this task to assess verbal memory included immediate recognition total correct words (VRMIRTC) pertaining to mean number of target words correctly recognized in addition to the number of distractor words correctly rejected during the immediate recognition phase, and free recall distinct stimuli (VRMFRDS) pertaining to the total number of distinct words – previously presented in the presentation phase – recalled correctly during the immediate free recall stage.

OTS: The outcome measures explored for the OTS task to evaluate executive function included mean choices to correct (OTSMCC), mean latency to correct choice (OTSMLC) (measured in unit of ms), and problems solved on first choice (OTSPSFC).

ERT: The outcome measures analyzed for this task to test for emotion recognition involved ERTTX (X standing for individual emotions i.e., H for happiness, SU for surprise) as the measure for total number of correct selections of the individual emotions across all assessed trials with a minimum of 0 and maximum of 15 selections; ERTMDCRTX and ERTOMDCRT evaluating the median latency for correct selections of individual or overall, for all emotions combined, respectively (measured in unit of ms).

This description is featured in table format in 2.1 Supplementary Tables.

### CANTAB Cambridge Cognition Website

Further details about the Cambridge Cognition cognitive assessment CANTAB can be viewed on Cambridge Cognition website (<https://cambridgecognition.com/digital-cognitive-assessments/>), including descriptions of Rapid Visual Information Processing – RVP (<https://cambridgecognition.com/rapid-visual-information-processing-rvp/>), Verbal Recognition Memory – VRM (<https://cambridgecognition.com/verbal-recognition-memory-vrm/>), One Touch Stockings of Cambridge – OTS (<https://cambridgecognition.com/one-touch-stockings-of-cambridge-ots/>), and Emotion Recognition Task – ERT (<https://cambridgecognition.com/emotion-recognition-task-ert/>).

## Hot Cognition

### Visual Stimuli Characteristics

### Slides featured four different images per quadrant in a 2x2 configuration while the image dimensions were either 590 by 460 pixels (15.5 by 12.2 cm) or 320 by 460 pixels (8.5 by 12.2 cm). Each slide was presented for a duration of 10.5 seconds, with 1 second intervals between slides showcased by a blank screen, culminating in a total of 15 minutes presentation time (Eizenman et al., 2003).

### KDEF Website

Additional information on KDEF images can be accessed on KDEF website (<https://kdef.se/home/aboutKDEF>).

# Supplementary Figures and Tables

## Supplementary Tables

| Measure Name | Measure Description | Description | Score Direction * | Units | Min | Max |
| --- | --- | --- | --- | --- | --- | --- |
| RVPPH | RVP Probability of Hit | Percentage of total number of target sequences correctly responded to, divided by total number of target sequences assessed (Correct hits ÷ Total number of sequences) | + | n/a | 0 | 1 |
| RVPA | RVP A′ | A′ is a measure of signal detection regarding participant’s sensitivity towards target sequence | + | n/a | 0 | 1 |
| RVPML | RVP Mean Response Latency | Mean response latency on trials where participant correctly responded | - | Ms | 100 | 1900 |

**Supplementary Table 1. RVP Outcome Measures**. Descriptions of chosen outcome measures of RVP task for analysis. Outcome measure highlighted in grey had normative data available and was recommended by CANTAB. * Score direction pertains to the directionality of the measure such that higher scores represent better (a positive (+) sense) or worse (a negative (-) sense) performance (Cambridge Cognition, 2019b).

| Measure Name | Measure Description | Description | Score Direction * | Units | Min | Max |
| --- | --- | --- | --- | --- | --- | --- |
| VRMIRTC | VRM Immediate Recognition: Total Correct | Total number of target words correctly recognized, plus total number of distractor words correctly rejected, in the immediate recognition phase | + | n/a | 0 | 36 |
| VRMDRTC | VRM Delayed Recognition: Total Correct | Total number of target words correctly recognized, plus total number of distractor words correctly rejected, in the delayed recognition phase | + | n/a | 0 | 36 |
| VRMFRDS | VRM Free Recall: Distinct Stimuli | Total number of distinct words previously presented in the presentation phase, correctly recalled during the immediate free recall stage | + | n/a | 0 | 18 |

**Supplementary Table 2. VRM Outcome Measures**. Descriptions of chosen outcome measures of VRM task for analysis. Outcome measures highlighted in grey were recommended by CANTAB. * Score direction pertains to the directionality of the measure such that higher scores represent better (a positive (+) sense) or worse (a negative (-) sense) performance (Cambridge Cognition, 2019b).

| Measure Name | Measure Description | Description | Score Direction * | Units | Min | Max |
| --- | --- | --- | --- | --- | --- | --- |
| ERTTH | ERT Total Hits | Total number of correct selections across all trials | + | n/a | 0 | 90 |
| ERTTHH | ERT Total Hits Happiness | Total number of correct “happiness” emotion selection across all trials | + | n/a | 0 | 15 |
| ERTTHS | ERT Total Hits Sadness | Total number of correct “sadness” emotion selection across all trials | + | n/a | 0 | 15 |
| ERTTHA | ERT Total Hits Anger | Total number of correct “anger” emotion selection across all trials | + | n/a | 0 | 15 |
| ERTTHF | ERT Total Hits Fear | Total number of correct “fear” emotion selection across all trials | + | n/a | 0 | 15 |
| ERTTHSU | ERT Total Hits Surprise | Total number of correct “surprise” emotion selection across all trials | + | n/a | 0 | 15 |
| ERTTHD | ERT Total Hits Disgust | Total number of correct “disgust” emotion selection across all trials | + | n/a | 0 | 15 |
| ERTOMDCRT | ERT Overall Median Correct Reaction Time | Overall median latency where participant correctly selected the emotion word, across all trials | - | ms | 0 | ∞ |

**Supplementary Table 3. ERT Outcome Measures**. Descriptions of ERT outcome measures chosen a priori for analysis. Outcome measure highlighted in grey was recommended by CANTAB * Score direction pertains to the directionality of the measure such that higher scores represent better (a positive (+) sense) or worse (a negative (-) sense) performance (Cambridge Cognition, 2019b).

| Measure Name | Measure Description | Description | Score Direction * | Units | Min | Max |
| --- | --- | --- | --- | --- | --- | --- |
| OTSMCC | OTS Mean Choices to Correct | Mean number of selections it took the participant before selecting correct choice, across all correct assessed trials | - | n/a | 1 | 7 |
| OTSPSFC | OTS Problems Solved on First Choice | Total number of assessed trials where participant selected correct choice on first attempt, across all assessed trials | + | n/a | 0 | 15 |
| OTSMLC | OTS Mean Latency to Correct | Mean latency until correct choice was selected, across all assessed trials | - | ms | 0 | ∞ |

**Supplementary Table 4. OTS Outcome Measures**. Descriptions of chosen outcome measures of OTS task for analysis. Outcome measure highlighted in grey had normative data available and was recommended by CANTAB. * Score direction pertains to the directionality of the measure such that higher scores represent better (a positive (+) sense) or worse (a negative (-) sense) performance (Cambridge Cognition, 2019b).

## Supplementary Figures

QIDS only

QIDS only

QIDS only

**Supplementary Figure 1.** Study Timeline Overview. Time points with * mark healthy control group participation.

**Supplementary Figure 2.** CONSORT Flow Diagram. The progression of participants involvement throughout the phases of the study from group allocation, to visit completion and analysis.


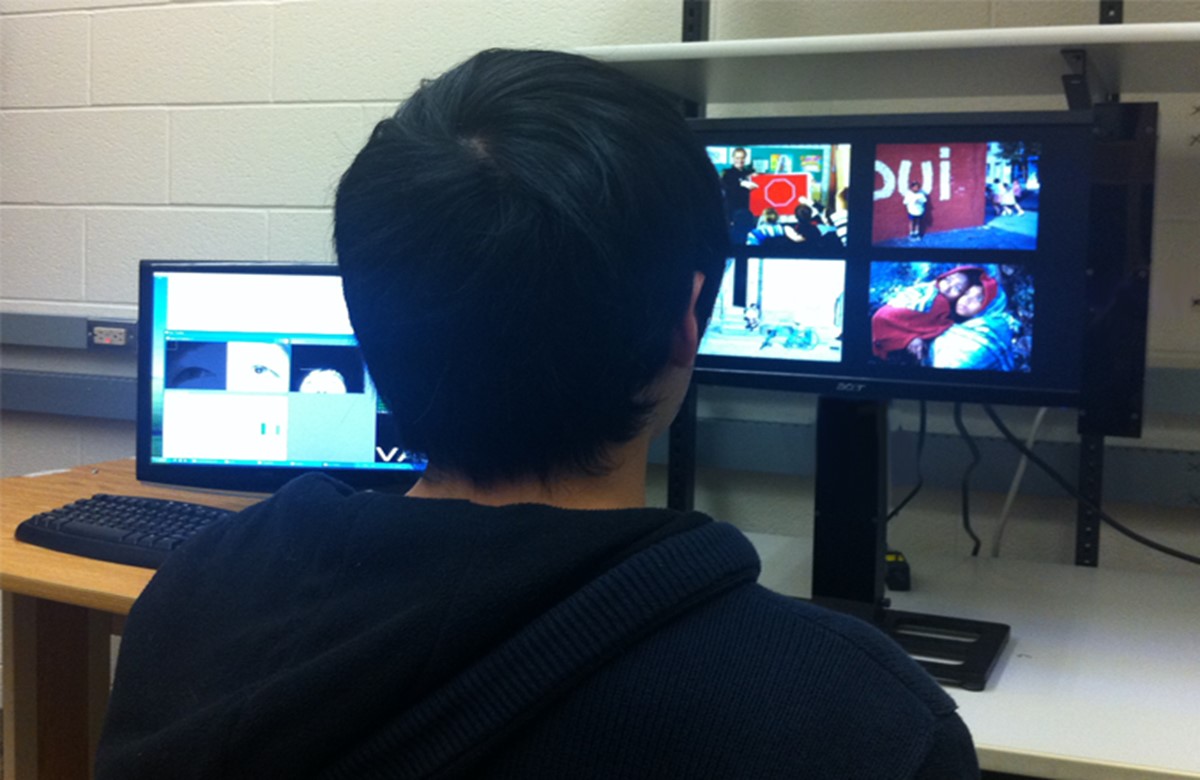


**Supplementary Figure 3.** Eye tracking apparatus developed by Eizenman and colleagues at EL-MAR Incorporation (EL-MAR Inc., Toronto, Ontario, Canada).


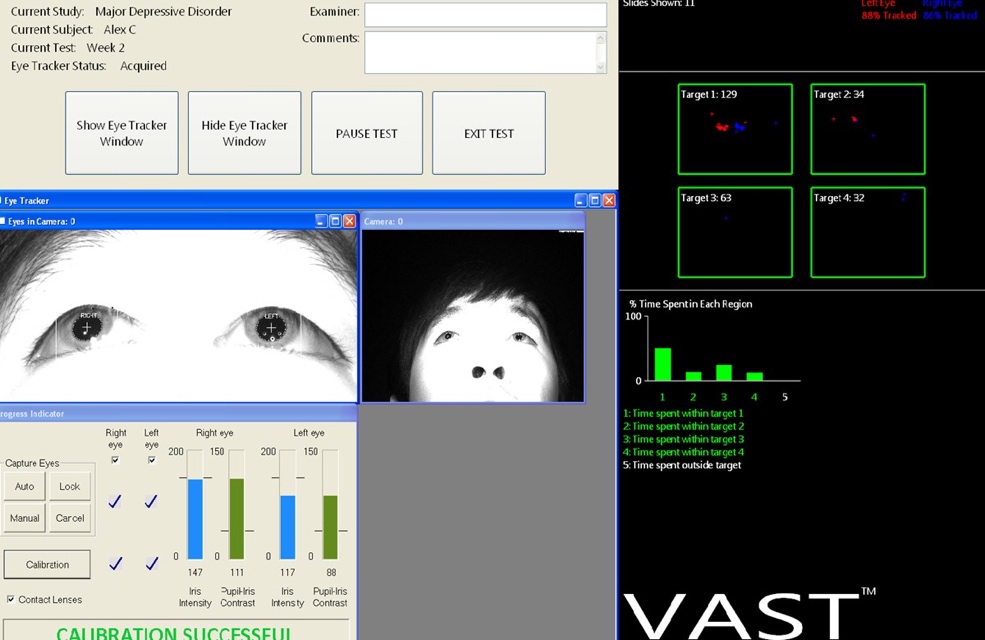


**Supplementary Figure 4.** Visual Attention Scanning Technology software (VAST, EL-MAR Inc., Toronto, Ontario, Canada) tracking eye gaze positions during the eye tracking task.


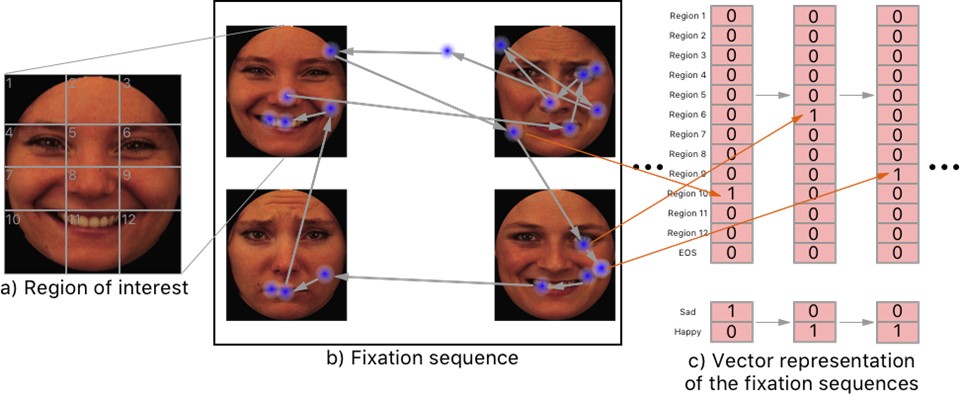


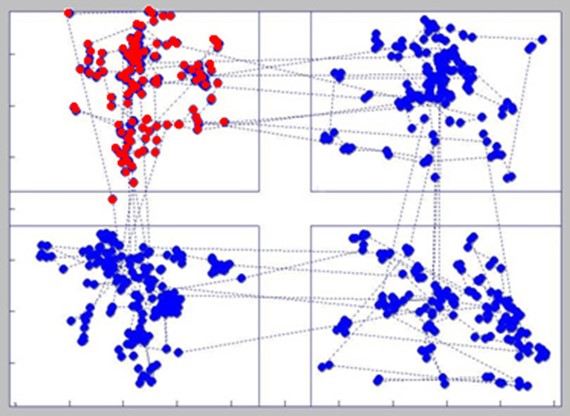


**Supplementary Figure 5.** Eye gaze position tracking on VAST Software.

**
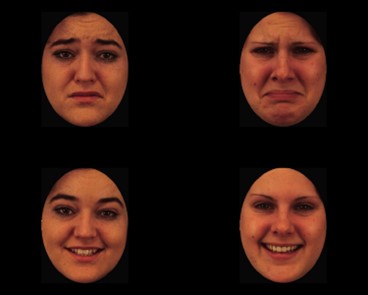
**

**Supplementary Figure 6.** KDEF Slide Example.
